# Enhancement of experimental cutaneous leishmaniasis by Leishmania extract: identification of a disease-associated antibody specificity

**DOI:** 10.1186/s13104-015-1158-0

**Published:** 2015-05-14

**Authors:** Virgínia M. G. Silva, Cíntia F. de-Araújo, Isabela C. Navarro, Pablo R. S. Oliveira, Lain Pontes–de-Carvalho

**Affiliations:** Centro de Pesquisas Gonçalo Moniz, Fundação Oswaldo Cruz, Salvador, BA 40296-710 Brazil; Universidade Estadual do Sudoeste da Bahia, Departamento de Ciências Biológicas, Jequié, BA 45206-190 Brazil; Escola Bahiana de Medicina e Saúde Pública, Salvador, BA 40050-420 Brazil

**Keywords:** *Leishmania*, Antibody response, Disease progression

## Abstract

**Background:**

Both *Leishmania braziliensis* and *Leishmania amazonensis* induce cutaneous disease when injected in the skin of BALB/c mice. However, *L. amazonensis* may also visceralize in that strain of mice, infecting mainly the liver and spleen. In addition, whereas BALB/c mice die with a progressive cutaneous disease when infected by *L. amazonensis*, the infection by *L. braziliensis* is spontaneously cured. In a previous work, we have found that intravenous injections of *L. amazonensis* amastigote extract (LaE) potentiated a *L. braziliensis* infection in BALB/c mice, and that this infection-promoting activity could be inhibited by the addition of protease inhibitors to the extract.

**Methods:**

In order to detect markers of disease evolution, in the present work we analyzed the specificity of the anti-*L. amazonensis* antibody response of L. braziliensis-infected BALB/c mice injected intravenously with saline or LaE, supplemented or not with protease inhibitors, by the Western blot technique.

**Results:**

IgG1 antibodies recognizing an antigen with apparent molecular weight of 116 kDa were specifically detected in BALB/c mice that had been turned susceptible to *L. braziliensis* infection by injections of LaE.

**Conclusion:**

A Th2 immune response (IgG1 antibody-producing) against this 116 kDa antigen, therefore, could be associated with susceptibility to severe *Leishmania* infection.

## Background

*Leishmania* parasites proliferate either as extracellular promastigotes, in the sand-fly vector, or as intracellular amastigotes, inside the phagolysosome of mammalian macrophages. Depending on the *Leishmania* species or isolate, and on the nature of the host immune response, the infection can cause distinct forms of disease, ranging from self-limiting cutaneous lesions to lethal visceral illness [1–3]. The leishmaniases are difficult to treat, and parasite resistance against the currently available drugs is increasing [4].

Although *Leishmania braziliensis* causes a serious health problem in South America [5], leading in some cases to mutilating nasal and/or oral lesions (reviewed in [6]), few experimental studies on the characterization of its antigens, and on the immune response against them, have been performed [7–9]. Contrasting to what can be observed in *L. braziliensis*-infected individuals, some *L. amazonenis*-infected individuals develop a progressive form of leishmaniasis, with multiple, heavily parasitized cutaneous nodules (diffuse cutaneous leishmaniasis), which is clearly associated with a failure of the patients’ immune system to mount a parasite-specific Th1 immune response [10, 11].

In the mouse, it has been shown that antibodies of the IgG1 isotype are produced mainly during a Th2 immune response, and IgG2a antibodies are produced mainly during Th1 immune responses [12]. In murine leishmaniasis models, the Th2 immune response has been associated with disease susceptibility, whereas the Th1 immune response has been associated to disease resistance [13]. Similarly to human beings, different species of *Leishmania* cause distinct diseases in mice. The genetic background of the mouse also affects the outcome of the infection [14–17].

A study on amastigote antigens, using the Western blot technique, demonstrated that antibodies from infected, healthy individuals and from leishmaniasis patients reacted with different parasites antigens [18]. Along the same line, a *Leishmania infantum* recombinant antigen, the k39, has been shown to discriminate antibodies from infected, asymptomatic individuals from those of patients with overt visceral leishmaniasis [19, 20]. Antibodies, therefore, may serve as markers of active disease in leishmaniasis. Prospective studies would need to be carried out in order to ascertain whether some antibodies produced by asymptomatic, *Leishmania*-infected individuals could be markers of resistance or susceptibility to the development of disease.

Our research group has shown that BALB/c mice, which are usually resistant to *L. braziliensis* [21], become susceptible if they are intravenously injected with a soluble extract of amastigotes of *Leishmania amazonensis* (and not of *L. braziliensis*), and that the supplementation of the extract with serine-protease inhibitors reduces this effect [22].

In order to identify possible antigenic markers of susceptibility to disease associated with the biologically active *L. amazonensis* extract, in the present work the specificity of the anti-*Leishmania* antibody response was assessed by Western blot in *L. braziliensis*-infected BALB/c mice intravenously injected with the *L. amazonensis* extract. The extract was supplemented or not with protease inhibitors and was injected one week before the infection and every two weeks thereafter. Antibodies against an antigen with an apparent molecular weight of 116 kDa were only detected in the sera of the mice injected with the biologically active extract (and not in the sera of the mice injected with the protease inhibitors-treated, biologically inactive extract).

## Methods

### Mice

Specific pathogen-free, 8-12 week-old, male BALB/c mice were maintained at the animal facilities of the Centro de Pesquisas Gonçalo Moniz, Fundação Oswaldo Cruz, Salvador, Bahia, and provided with rodent diet and water *ad libitum*. All procedures performed on the animals were approved (protocol number 004/2004) and conducted in accordance with norms of the Ethical Committee in Laboratory Animal Utilization of the Centro de Pesquisas Gonçalo Moniz, Fundação Oswaldo Cruz, Salvador, Brazil.

### Parasites and amastigote extract

The MHOM/Br87/Ba125 *L. amazonensis* and MHOM/Br/3456 *L.braziliensis* strains were used. Their infectivities were maintained by regular inoculations of promastigotes in susceptible BALB/c mice and golden hamsters, respectively. Promastigotes, derived from tissue amastigotes, were cultured at 23 °C in Schneider’s medium (Sigma Chemical Co., Saint Louis, MO, USA), pH 7.2, supplemented with 50 μg/mL of gentamycin and 10 % of heat-inactivated fetal bovine serum (FBS; HIFCS, Gibco Laboratories, Grand Island, NY, USA) for *L. amazonensis*, or 20 % FBS for *L. braziliensis. L. amazonensis* axenic amastigotes were obtained by the differentiation of stationary-phase promastigotes in axenic cultures. They were left to differentiate in Falcon flasks containing 2 × 10^7^ promastigotes/mL in Schneider’s medium supplemented with 5 % fetal bovine serum, pH 5.4, at 32 °C and 5 % CO_2_. After 5 days, more than 98 % of cells were morphologically undistinguishable from amastigotes, as described elsewhere [23]. The amastigotes were washed three times in ice-cold sterile saline, resuspended in ice-cold saline and lysed by exposition to ultrasound (10 times for 1 min on ice). The lysates were centrifuged at 16,000 g for 10 min at 4 °C, the supernatants filtered on membranes with 0.22 μm-diameter pores (Millipore, São Paulo, Brazil) and immediately stored at -70 °C in small aliquots. These filtered saline supernatants are called in this report *L. amazonensis* extract (LaE). They were shown to be free of bacterial endotoxin by the *Limulus* amebocyte enzyme assay (Biowittaker, MD, USA), and their protein content was determined by Lowry’s method [24]. Part of the prepared LaE was supplemented with protease inhibitors (10 mM phenylmethanesulfonyl fluoride, 5.7 mM N-p-tosyl-L-phenylalanine chloromethy l kNap-tosyl-L-lysyl chloromethyl ketone etone, 5.4 mM N-p-tosyl-L-lysine chloromethyl ketone hydrochloride, and 5.9 mM 4-nitrophenyl hepta-O-acetyl-1-thio-beta-lactoside; Sigma Chemical Co., St Louis, MO, USA).

### Injection of mice with *Leishmania* extract

Animals from groups of 4 to 5 BALB/c mice received four 0.2-mL i.v. injections of either LaE or LaE with protease inhibitors. The injections were separated by an interval of two weeks, starting one week before the mice were infected as described below. Each injection contained 200 μg of protein. Control groups of mice received four 0.2-mL injections of either saline or saline plus protease inhibitors.

### Murine model of cutaneous leishmaniasis

Ten million *L. braziliensis* promastigotes, obtained from stationary-phase culture, were subcutaneously inoculated into one of the hind footpads of BALB/c mice, seven days after the first injection of the LaE. The hind pad thicknesses were weekly measured with a digital caliper and the lesion sizes estimated by subtracting the thickness of the uninfected pad from the thickness of the infected pad. Tissue parasitism was estimated as described below.

### Evaluation of parasite load

Parasite loads in the footpads were estimated by limiting dilution [25] six weeks after infection. Briefly, the infected footpads were macerated in Schneider’s medium and centrifuged at 50 g for 10 min, at 4 °C. The supernatants were re-centrifuged at 1540 g for 10 min at 4 °C and the pellets were resuspended in Schneider’s medium, supplemented with 50 μg.mL^−1^ of gentamycin, and 20 % FBS. The suspension was serially diluted in 10-fold dilutions and distributed in triplicates in 96-well culture plates. The number of viable parasites in each footpad was estimated from the reciprocal of the highest dilution at which promastigotes could be detected after a 7-day culture at 23 °C, and was expressed as parasites per milligram of tissue.

### Determination of antibody specificity by Western blot

Sodium dodecyl sulfate-polyacrylamide gel electrophoresis (SDS-PAGE) of LaE was performed on 12 % polyacrylamide gels using a Mini Protean II apparatus (Bio-Rad, California, USA). Wells were loaded with LaE containing 20 μg/mL of protein, resuspended in SDS-sample buffer and boiled for 4 min, and the electrophoresis carried out as described elsewhere [26]. Proteins were electrophoretically transferred from the gel to nitrocellulose membranes. The nitrocellulose membranes were cut into vertical strips and blocked for 12 h with 0.15 M phosphate-buffered saline, pH 7.2 (PBS) containing 10 % FBS, at 4 °C. Incubation with mouse sera (diluted 1:1000 in PBS containing 0.05 % of Tween 20 and 10 % of FBS) was carried out during 1 h at room temperature with mechanical agitation. After five 1-min washes in PBS, the membranes were incubated during 1 h with appropriately diluted horseradish peroxidase-conjugated anti-mouse IgG1 or IgG2a (Sigma Chemical Co., Saint Louis, MO, USA) in PBS containing 0.05 % Tween 20 and 5 % FBS, at room temperature. The nitrocellulose strips were finally incubated with a mixture of 3’3-diaminobenzidine (Sigma Chemical Co., Saint Louis, MO, USA) and H_2_O_2_ in PBS. Naïve mouse sera were used as negative controls.

### Statistical analysis

When the distribution of the data was found to be Gaussian by the D’Agostino and Pearson’s method, comparisons among the groups were performed by ANOVA followed by the Newman-Keuls’s method. When the distribution was found to be non-Gaussian, comparisons among the groups were performed by the Kruskal-Wallis method followed by the Dunn’s post test. Results were considered significant when *P* ≤ 0.05. The GraphPad Prism program, version 5, was used to perform the calculations.

## Results

### Lesion size and parasite burden in experimentally infected mice

In a previously published work, we have shown that injection of LaE together with protease inhibitors abolished the enhancing effect of the LaE on the *Leishmania* infection in BALB/c mice [22]. We, therefore, have analysed in the present work not only the antibodies present in the sera of saline-injected mice, but also the antibodies present in the sera of a group of mice that had been injected with LaE plus protease inhibitors.

**Fig. 1:**
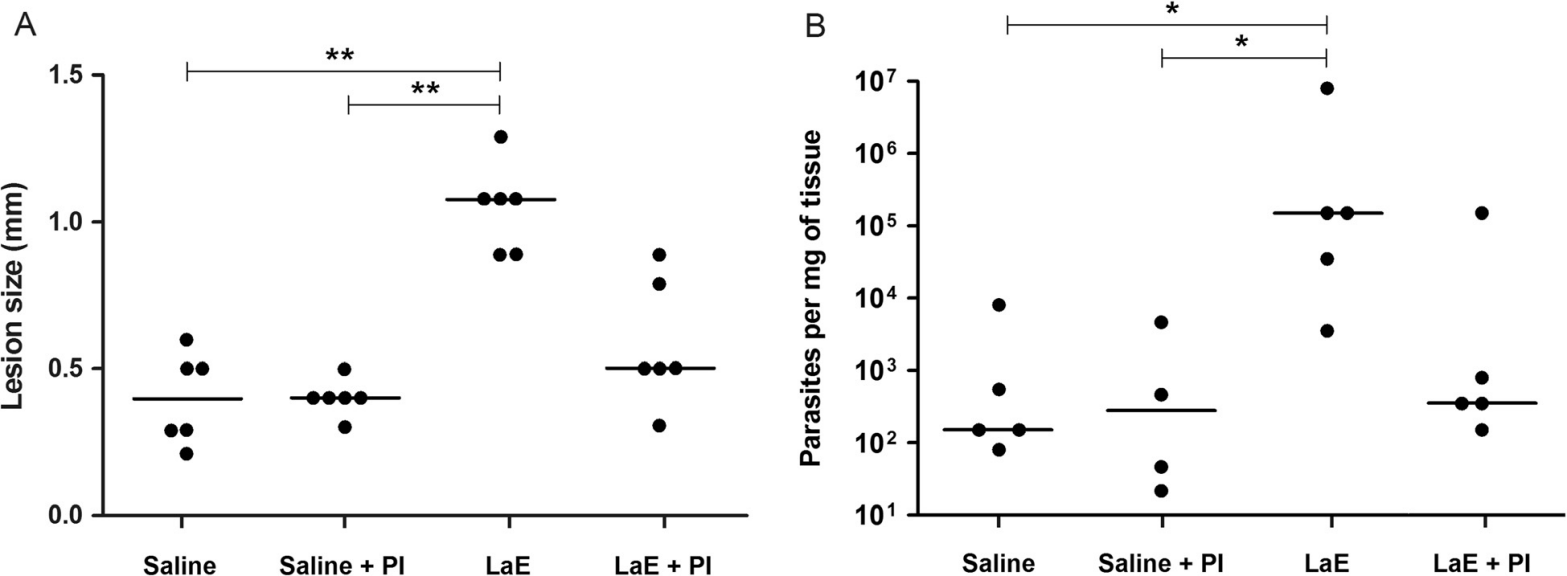
Lesion size and parasitism in mouse footpads six weeks after infection. The mice were treated with saline (Saline), saline supplemented with protease inhibitors (Saline + PI), or with *L. amazonensis* extract in the presence (LaE + PI) or absence (LaE) of protease inhibitors, as detailed in the Materials and Methods. **a** Lesion sizes, measured with a digital caliper as described in the Material and Methods. **b** Parasite numbers per milligram of tissue, as assessed by limiting dilution. Each symbol represent the value obtained from an individual mouse. The horizontal lines represent the median values of the results. *P ≤ 0.05; **P ≤ 0.01 (Kruskal-Wallis’s test followed by Dunn’s post test)

As in previously published work, the four biweekly intravenous injections of LaE significantly increased the size of the lesions caused by *L. braziliensis* in BALB/c mice (Fig. 1a), whereas the group of mice that received LaE in the presence of protease inhibitors did not differ significantly from the control group (mice that received saline; Fig. 1a).

The number of parasites in the foot pad lesion of BALB/c mice, infected with *L. braziliensis*, that received four biweekly LaE i.v. injections, was significantly higher than those in animals injected with saline (Fig. 1b). The infected footpads of the mice injected with protease inhibitors-treated LaE had on average 100-fold fewer parasites than the footpads of the mice injected with untreated LaE. The group of mice that received LaE in the presence of protease inhibitors did not differ significantly from the control group in terms of parasite load (Fig. 1b).

Although differences between the groups of mice treated with LaE and LaE plus protease inhibitors, both in terms of lesion size and tissue parasitism, could be clearly seen (Fig. 1), they were not statistically significant.

### Specificity of the antibody response against *L. amazonensis* amastigote antigens

**Fig. 2:**
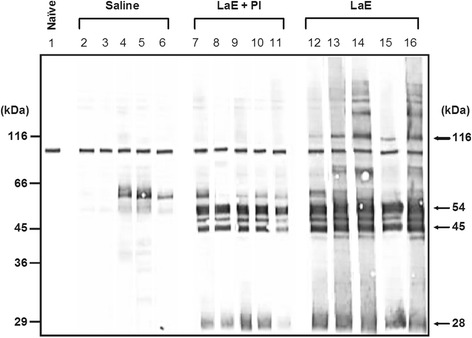
Reactivity against *L. amazonensis* antigens, as assessed by Western blot, of IgG1 antibodies in the sera of BALB/c mice that had been injected with *L. amazonensis* extract and infected with *L. braziliensis*. The sera were from blood samples collected five weeks after infection. The infected mice were treated with saline (Saline; lanes 2 to 6), *L. amazonensis* extract supplemented with protease inhibitors (LaE + PI, lanes 7 to 11) or unsupplemented *L. amazonensis* extract (LaE, lanes 12 to 16), as detailed in the Materials and Methods. The result obtained with the serum of a naïve mouse is shown in lane 1. The positions of molecular weight markers are shown on the left of the figure

One protein with apparent molecular weight of 28 kDa, and three proteins with apparent molecular weights from 45 to 54 kDa were only detected by the IgG1 antibodies from mice injected with LaE, regardless of its supplementation with protease inhibitors (Fig. 2). Additional antigens were recognized only by the sera of the mice treated with the unsupplemented extract. These sera stained from 6 to 12 or more bands (Fig. 2, lanes 12 to 16). On the other hand, the sera of the mice injected with protease inhibitor-supplemented extract specifically stained only 4 or 5 bands (Fig. 2, lanes 7 to 11). An antigen with apparent molecular weight of 116 kDa was recognized by all sera of the mice injected with unsupplemented LaE (Fig. 2, lanes 12 to 16), and by none of the sera from the mice injected with the protease inhibitor-supplemented extract (Fig. 2, lanes 7 to 11). The recognition pattern of the serum from a particular mouse injected with unsupplemented extract (Fig. 2, lane 15) only differed from the recognition patterns of the sera from mice treated with the protease inhibitor-supplemented extract (Fig. 2, lanes 7 to 11) by the recognition of that particular antigen.

**Fig. 3:**
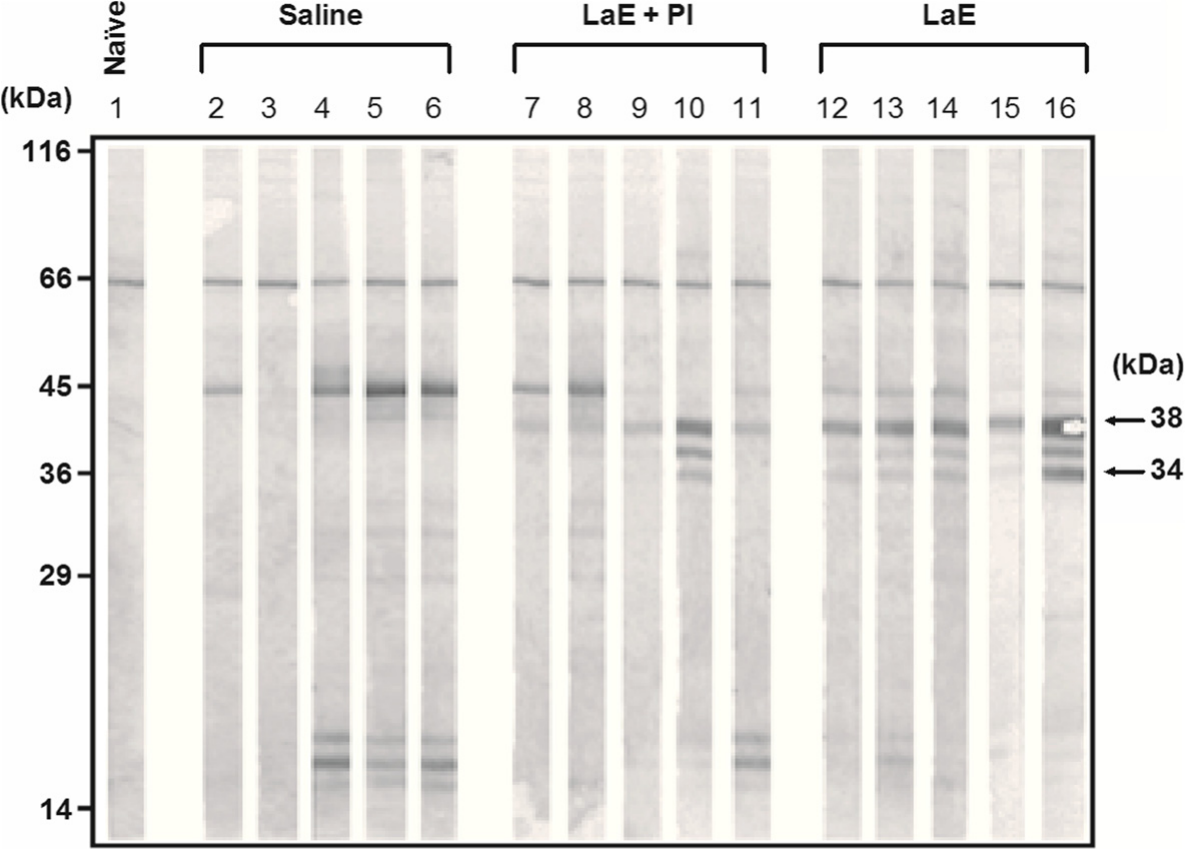
Reactivity against *L. amazonensis* antigens, as assessed by Western blot, of IgG2a antibodies in the sera of BALB/c mice that had been injected with *L. amazonensis* extract and infected with *L. braziliensis*. Sera were from blood samples collected five weeks after infection. The infected mice were treated with saline (Saline; lanes 2 to 6), *L. amazonensis* extract supplemented with protease inhibitor (LaE + PI, lanes 7 to 11) or unsupplemented *L. amazonensis* extract (LaE, lanes 12 to 16), as detailed in the Materials and Methods. The result obtained with the serum of a naïve mouse is shown in lane 1. The positions of molecular weight markers are shown on the left of the figure

Similar antigens were recognized by IgG2a antibodies of the *L. braziliensis*-infected BALB/c mice, injected or not with LaE, supplemented or not with protease inhibitors (Fig. 3). Three antigens with apparent molecular weights ranging from 34 to 38 kDa reacted with IgG2a antibodies only from the sera of mice treated with LaE, supplemented or not with protease inhibitors (Fig. 3). No difference in antigen recognition was observed with IgG2a antibodies from animals that received unsupplemented LaE and animals that received LaE supplemented with protease inhibitors (Fig. 3, lanes 7 to 16).

## Discussion

The transformation from promastigotes to amastigotes provides the necessary adaptations for the *Leishmania* to survive in the mammalian environment, including mechanisms to evade or subvert the host immune response, such as the escape to a safe, protease-free intracellular vacuole and the production of immunomodulatory molecules [27–29]. In this study, the presence of a *L. braziliensis* infection-enhancing activity in soluble extracts of *L. amazonensis* amastigotes, as observed previously in our laboratory [22, 30], was confirmed: larger numbers of parasites were found in the foot pad of *L. braziliensis*-infected BALB/c mice that received LaE than in those from animals injected only with saline (Fig. 1). In the present work, the LaE was injected in a relatively large amount by the intravenous route. In a previously published work [30], similar results were obtained when the intradermal route was used.

It has been reported that the injection of LaE together with protease inhibitors reverts its infection-enhancing effects [22]. In fact, it has been shown that *Leishmania amazonensis* serine proteases may be disease-aggravating components of a crude anti-leishmaniasis vaccine [31]. Thus, in the present work we compared the specificity of the humoral immune response of the mice injected only with LaE (susceptible to *L. braziliensis* infection) with that of the response of mice injected with a mixture of LaE and protease inhibitors (relatively resistant to the development of *L. braziliensis* infection).

As shown herein, the nature of the humoral immune response against *L. amazonensis* antigens differed in *L. braziliensis*-infected mice rendered susceptible by the intravenous injection of LaE from those of relatively resistant mice (injected with LaE supplemented with protease inhibitors or with saline). Quite interestingly, these differences were most evident for IgG1 antibodies (Fig. 2), and not for IgG2a antibodies (Fig. 3), a fact which is consistent with the LaE acting through the promotion of a Th2-dependent immune response. This confirm previous results showing increased production of IL-4 by lymph node cells of LaE-injected mice and the absence of infection-enhancing effect of this extract in IL-4-knockout mice [22].

The observed differences in the production of IgG1 antibodies were both quantitative (antibodies of infected BALB/c mice injected only with saline, or even with protease inhibitor-supplemented LaE, reacted with fewer peptides than antibodies from mice injected with unsupplemented LaE), and qualitative: a 116 kDa protein band of *L. amazonensis* amastigotes was exclusively recognized by sera of unsupplemented LaE-treated BALB/c mice. It is possible that the presence of IgG1 antibodies against one or more of these antigens could be a marker of an infection-promoting immune response induced by *L. amazonensis*. These antigens could in fact lead to the generation and maintenance of a predominant Th2 immune response that would hinder an otherwise effective Th1 response, allowing the progression of the disease. It would be interesting to find out if sera from patients with diffuse cutaneous leishmaniasis differ from those of patients with localized cutaneous leishmaniasis caused by *L. amazonensis* by the presence of antibodies against these antigens.

A Th2 immune response against the protein with apparent MW of 116 kDa might be one of the many escape mechanisms that have evolved during the thousands of years of interaction of the parasite with the mammalian hosts’ immune system. For instance, other *Leishmania* molecules, such as the kinetoplastid membrane protein 11 (KMP-11) [32], the *Leishmania*-homologue of activated C kinase (LACK) [33], the lipophosphophoglycan [34] and the Gp63 [35] have been shown, in different situations, either *in vitro* or *in vivo*, to enhance the *Leishmania* infection.

The expression of a serine protease with an apparent molecular weight of 115 kDa by *L. amazonensis* has been described by Silva-Lopez and collaborators [36]. In an electrophoretic-zymographic study performed by our research group, a protein with the same apparent molecular weight and with protease activity was found in a *L. amazonensis* extract and not in a *L. braziliensis* extract (Oliveira & Navarro, unpublished data). It is possible that this serine protease is the protein recognized only by antibodies from mice with severe leishmaniasis, which had been injected with LaE without serine protease inhibitors in the present work. *L. amazonensis* serine protease activity has been shown to enhance the *Leishmania* infection, but the responsible protein has not been identified [22, 32].

Another interesting fact observed in the present work was that the untreated *L. braziliensis*-infected animals produced antibodies, mainly of the IgG2a isotype, recognizing cross-reactive *L. amazonensis* antigens (Fig. 3, columns 1-5). This is in accordance to the fact that these animals preferentially produce a Th1-dependent immune response [22, 32]. These cross-reactive antibodies could be useful for identifying, in *Leishmania* DNA libraries, genes encoding recombinant proteins able to elicit potentially protective Th1 immune responses.

It is possible that antibodies against the 116 kDa protein are produced during the human infection and could be used as markers of disease susceptibility. This possibility is amenable to investigation by seroepidemiological studies.

## Conclusions

In summary, we investigated the pattern of antigen recognition by antibodies from BALB/c mice that were rendered susceptible to *L. braziliensis* infection by intravenous injections of a *L. amazonensis* extract. We found that a protein with a molecular weight of approximately 116 kDa was specifically recognised by antibodies from those mice. No protein with this molecular weight possessing infection-enhancing activity has been described in the literature so far. The immune response against this protein, therefore, might be detrimental to the development of a protective immune response.

## Abbreviations

LaE: *L. amazonensis* amastigote extract
PI: Protease inhibitors
